# Oral health and orofacial pain in older people with dementia: a systematic review with focus on dental hard tissues

**DOI:** 10.1007/s00784-016-1934-9

**Published:** 2016-09-08

**Authors:** Suzanne Delwel, Tarik T. Binnekade, Roberto S. G. M. Perez, Cees M. P. M. Hertogh, Erik J. A. Scherder, Frank Lobbezoo

**Affiliations:** 1 Faculty of Behavioral and Movement Sciences, Department of Clinical Neuropsychology, VU University, Amsterdam, The Netherlands; 2 Faculty of Dentistry, Department of Oral Kinesiology, Academic Centre for Dentistry Amsterdam (ACTA), University of Amsterdam and VU University Amsterdam, MOVE Research Institute Amsterdam, Amsterdam, The Netherlands; 3 Department of Anesthesiology, EMGO+ Institute for Health and Care Research, VU University Medical Centre Amsterdam, Amsterdam, The Netherlands; 4 Faculty of Medicine, Department of Elderly Care Medicine, VU University Medical Centre Amsterdam, Amsterdam, The Netherlands

**Keywords:** Dementia, Elderly, Aged, Gerodontology, Facial pain, Oral health, Stomatognathic disease

## Abstract

**Objective:**

The aim of this review was to provide a systematic overview including a quality assessment of studies about oral health and orofacial pain in older people with dementia, compared to older people without dementia.

**Methods:**

A systematic literature search was performed in PubMed, CINAHL, and the Cochrane Library. The following search terms were used: dementia and oral health or stomatognathic disease. The quality assessment of the included articles was performed using the Newcastle-Ottawa Scale (NOS).

**Results:**

The search yielded 527 articles, of which 37 were included for the quality assessment and quantitative overview. The median NOS score of the included studies was 5, and the mean was 4.9 (SD 2.2). The heterogeneity between the studies was considered too large to perform a meta-analysis. An equivalent prevalence of orofacial pain, number of teeth present, decayed missing filled teeth index, edentulousness percentage, and denture use was found for both groups. However, the presence of caries and retained roots was higher in older people with dementia than in those without.

**Conclusions:**

Older people with dementia have worse oral health, with more retained roots and coronal and root caries, when compared to older people without dementia. Little research focused on orofacial pain in older people with dementia.

**Clinical relevance:**

The current state of oral health in older people with dementia could be improved with oral care education of caretakers and regular professional dental care.

## Introduction

During recent decades, an improvement in oral health care was seen, and consequently, an increase in the number of remaining teeth at higher ages [1]. Aging is an important risk factor in the development of medical conditions [2], and general health has a wide-ranging interaction with oral health [3–12]. Therefore, with the aging of the population, an increase in oral health problems is to be expected.

Oral health in older people has been described in several studies, examining the number of teeth present, dentures, oral disease, and caries. Edentulousness is prevalent among older people all over the world and is highly associated with socio-economic status [1]. Dentures are particularly frequent among older people in the developed countries [4]. In these countries, full dentures in both the upper and lower jaw are worn by one third to half of the older population, while partial dentures or full dentures in one jaw are worn by three quarters of the older population [3]. Dental caries is highly prevalent in older people in several countries, such as Australia and the USA [5, 6] and is closely associated with social and behavioral factors [3, 6, 7]. More specifically, caries tends to be more prevalent in people with low income, irregular dentist visits, lower frequency of brushing teeth, and high sugar consumption [7–9]. The caries increments of older people (between 0.8 and 1.2 newly affected tooth surfaces per year) exceed that of adolescents (between 0.4 and 1.2 newly affected tooth surfaces per year) [6]. Altogether, older people have more oral health problems than younger adults, and also orofacial pain is considered to increase with age in the general population [10].

Oral health problems become even more prevalent in older people with dementia; as the disorder progresses, cognition, motor skills, and self-care decline, increasing the risk of oral health problems [11, 12]. Even though an increasing interest in oral health in older people with dementia is seen in recent years, an up-to-date review of literature, comparing oral health in older people with and without dementia, is lacking. Furthermore, a review of orofacial pain in older people with dementia is lacking entirely, while oral health problems can be an important cause of orofacial pain and discomfort. Consequently, the aim of this review was to provide a systematic overview including a quality assessment of studies about the oral health and orofacial pain of older people with dementia, compared to older people without dementia. For this review, the focus was on health of dental hard tissues and orofacial pain, representing the following available data: percentages of people with orofacial pain, edentulousness and dentures, the Decayed Missing Filled Index, number of teeth present and retained roots, and number of teeth with coronal and root caries. The health of oral soft tissues will be reviewed in a separate article.

## Methods

### Search, study selection, and quality assessment

A literature search was performed on March 31, 2016 in the following electronic databases: PubMed, CINAHL, and the Cochrane Library. In PubMed, the following search query was used: ((((“Oral Health”[Mesh] OR “Oral Health” [tiab])) OR (“Stomatognathic Diseases”[Mesh])) AND ((“Dementia”[Mesh] OR “Dementia”[tiab])). In CINAHL and the Cochrane Library, the same search terms were used, with database queries adjusted to the specific database. No restrictions with regard to language, year of publication, or methodology were applied during the search in order to maximize the inclusion of appropriate articles. Articles published in languages other than Dutch, English, and German were assessed by native speakers with dental knowledge for that particular language. Next, the titles, abstracts, and full texts were reviewed according to inclusion and exclusion criteria. The inclusion criteria were as follows: older people with dementia, oral health, stomatognathic disease, facial pain, and useable data. Exclusion criteria were as follows: age below 60, no dementia, not about oral health or stomatognathic disease, case report, review, and no useable data (e.g., no quantitative data).

The screening of the titles, abstracts, and full texts, as well as the assessment of the quality of the Dutch, English, and German studies, was done independently by a dentist (SD) and a neuropsychologist (TB). The criteria were formulated in advance, and disagreements between reviewers were resolved by consensus. Articles published in other languages were screened and assessed by a native speaker (for the particular language) with a background in dentistry. The reference lists of the included articles were scanned for complementary studies. If full texts were not available, or the dementia diagnosis or oral health data was unclear, the original authors were contacted up to a maximum of three times. If the dementia diagnosis or oral health data remained unclear, the article was excluded. The quality of the remaining articles, including risk of bias, was assessed with the Newcastle-Ottawa Scale (NOS), using a maximum score of 9 [13]. In this review, a NOS quality score of 7 (=78 % of the maximum score) or more, was considered a high score.

### Data extraction

Although the search focused on oral health in general, this review only discusses the dental hard tissue variables. The oral soft tissue variables will be reported in a separate review. The division between dental hard and soft tissues is often seen in articles that report oral health in older people with dementia [5, 14–16]. The first review author (SD) extracted the data from the included studies, and the second (TB) and last author (FL) checked the extracted data. The following data were extracted from the included articles: (1) study design (e.g., cross-sectional, case-control, cohort study); (2) participant characteristics (including age, dementia diagnosis, subtype, and severity); and (3) outcome measures, including orofacial pain, dentures, edentulousness, number of teeth present [17], decayed missing filled teeth (DMFT) index [18], coronal caries, root caries, and retained roots. If a study published baseline and follow-up data within the same article, only the baseline data was used. The principal summary measures used were percentages and means, including standard deviation. The heterogeneity of the data was checked.

## Results

### Study selection, characteristics, and participants

**Fig. 1:**
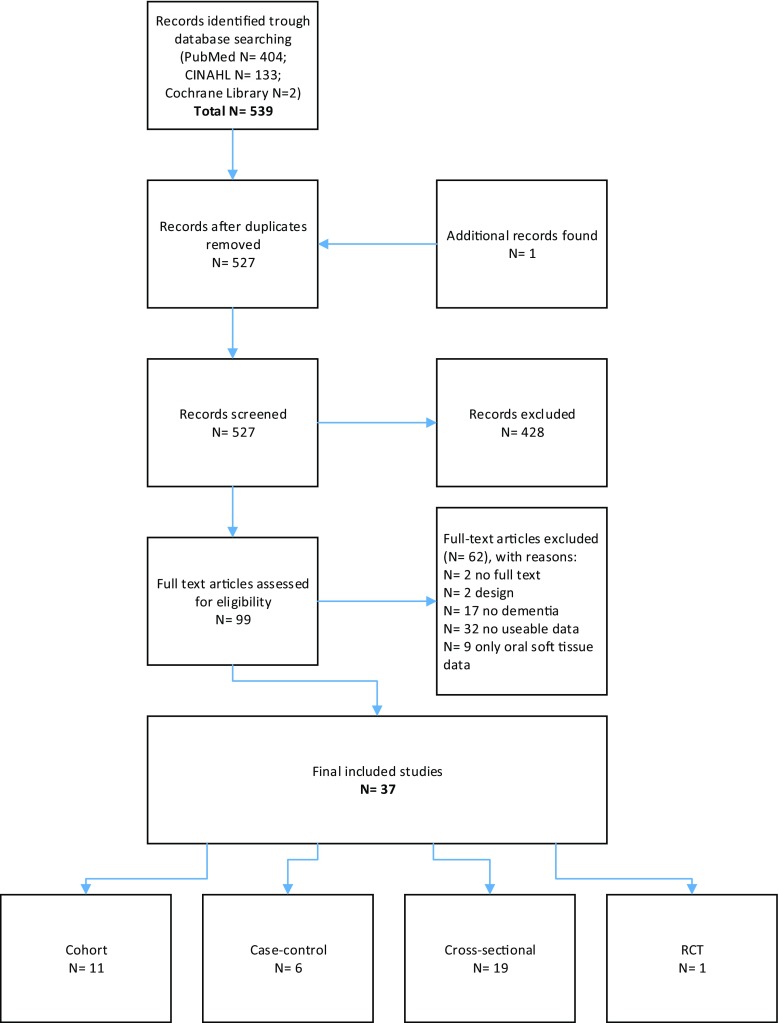
Flow chart of the literature search

The search yielded 577 studies, up to publication year 2016. After the duplicates had been removed, 527 studies remained. The titles and abstracts of the remaining studies were screened, leading to the exclusion of 428 studies because they did not meet the inclusion criteria. The 99 remaining full text articles were then examined for eligibility, of which 62 were then excluded because they did not meet the inclusion criteria. Only one study was added through scanning the reference lists of the included articles [19]. Thereafter, the quality of the 37 included studies was assessed. The flowchart of search is presented in Fig. 1. During the review process, 11 authors were contacted for further information of which seven replied. Additional information about the dementia diagnosis was given by Chen et al. and Del Brutto et al. [20–23] and additional data was provided by authors of Bomfim et al., Fjeld et al., Kersten et al., Lee et al., and Stewart et al. [24–27].

Of the final 37 included studies (Table 1), 11 were cohort studies, 6 were case-control studies, 19 were cross-sectional studies, and 1 had an randomized controlled trial (RCT) design. Most of the studies were in English; the articles of Nishiyama et al. and Sumi et al. were in Japanese [50, 55]. The relevant information of these two Japanese studies was extracted by a native Japanese speaker with dental knowledge; the study of Nishiyama et al. was excluded for not involving older people with dementia.

**Table 1:** Overview of studies about the health of dental hard tissues in older people with and without dementia

Study	Design	Dementia group (=*N*)	Mean age In years (SD)	Control group (=*N*)	Mean age in years (SD)	Dementia measure	Oral health measure hard dental tissues
Adam and Preston [28], UK	Cross-sectional	81 MoD-SeD	80.8 (7.63)	54 ND or MiD	85.5 (7.56)	Abbreviated Mental Test	Orofacial pain, dentures, edentulousness, DMFT
Bomfim et al. [24], Brazil	Cross-sectional	mv	mv	mv	mv	MMSE, chart, ADL	Present teeth, dentures
Chalmers et al. [14], Australia	Longitudinal cohort	116	<79: 78.4 % 80+: 21.6 %	116	<79: 78.4 % 80+: 21.6 %	MMSE	Present teeth, DMFT, root caries
Chalmers et al. [15], Australia	Longitudinal cohort	103	<79: 79.6 % 80+: 20.4 %	113	<79: 77.9 % 80+: 22.1 %	MMSE	Present teeth, dentures, DMFT, coronal caries, root caries
Chapman and Shaw [30], Australia	Cross-sectional	85 AD	74.9	–	–	Not described	Present teeth, dentures, DMFT
Chen et al. [22], USA	Cross-sectional	51 community 18 assisted living 501 NHR	79.3 (8.0) 80.9 (12.6) 82.6 (9.6)	–	–	Chart, ICD-9	Present teeth, decay or retained roots
Chu et al. [32], China	Case-control	59	79.8 (7.4)	59	79.8 (7.4)	Chart	DMFT
Cohen-Mansfield [33], USA	Cross-sectional	21	88.0 (mv)	–	–	MMSE, MDS-COGS	Broken or fractured teeth, caries, dentures, retained roots
De Souza Rolim et al. [34], Brazil	Case-control	29	75.2 (6.7)	30	61.2 (11.2)	NINCDS-ADRDA for AD, MMSE	Orofacial pain, DMFT
De Souza Rolim et al. [34], Brazil	Case-control	29		–	–	NINCDS-ADRDA for AD, MMSE	Orofacial pain, DMFT
Ellefsen et al. [35], Denmark	Cross-sectional (baseline)	61 AD 26 OD	82.8 (5.7) 81.5 (4.8)	19	79.8 (7.3)	ICD-10	Coronal caries, root caries
Ellefsen et al. [36], Denmark	Cohort (baseline, follow-up)	49 AD 15 OD	83.6 (5.5) 81.3 (4.0)	13	79.9 (7.7)	ICD-10	Present teeth, DMFT, CCI, NCI, ADJCI
Ellefsen et al. [38], Denmark	Cross-sectional (follow-up)	61 AD	82.8 (5.7)	–	–	ICD-10	Present teeth, DMFT, coronal caries, root caries
Elsig et al. [39], Switzerland	Cross-sectional	29	82.5 (6.3)	22	81.9 (6.5)	NP, MMSE, CERAD, CDR	Present teeth
Eshkoor et al. [40] Malaysia	Cross-sectional	1210	71.0 (7.38)	–	–	MMSE	Presences of teeth or dentures
Fjeld et al. [25], Norway	RCT	159	85.5 (7.7)	43	88.5 (6.6)	Evaluated by physician	Present teeth
Furuta et al. [41], Japan	Cross-sectional	143 MiD-MoD 61 SeD		82		CDR	Present teeth, dentures
Hatipoglu et al. [42], Turkey	Prospective cohort	31 AD	67.6 (9.14)	47	65.3 (7.0)	MMSE	Dentures, DMFT
Jones et al. [43], USA	Cohort	23	67.4 (7.5)	46	66.1 (6.9)	Longitudinal study of dementia	Present teeth, CCI, RCI
Kossioni et al. [44], Greece	Case-control	27	76.5 (6.8)	84		DSM-IV	Present teeth, DMFT
Lee et al. [26], USA	Cross-sectional	19 MiD	MiD 83.9 (7.9)	169	77.4 (5.8)	MCI, MiD: DSM-IV	Missing teeth, coronal caries, root caries
Luo et al. [45], China	Cross-sectional	120	80.9 (7.4)	2389	70.0 (7.7)	DSM-IV	Missing teeth
Minakuchi et al. [46], Japan	Cross-sectional	155		50		COD by MHLW JP	Present teeth, dentures
Nordenram et al. [47], Sweden	Case-control	40	87.0 (7.0)	40	87.0 (6.6)	DSM-III-R, MMSE	Present teeth, dentures
Philip et al. [16], Australia	Cross-sectional	84	85.7 (9.6)	102	84.3 (9.9)	Chart, ADLOH	DMFT, retained roots
Ribeiro et al. [48], Brazil	Cross-sectional	30	79.1 (5.6)	30	67.8 (5.5)	ICD-10, DSM-IV, MMSE, CDR	Present teeth, DMFT, dentures
Ship and Puckett [12], USA	Cohort	21	64.0 (9.0)	21	65.0 (12)	NINCDS-ADRDA CT, MRI, PET, NP	Present teeth, DMFT
Srilapanan et al.[50], Thailand	Cross-sectional	69	75.5 (7.0)	0	–	Chart, MMSE	Dental habits, present teeth, dentures, DMFT, caries
Sumi et al. [50], Japan	Cohort	10	77.7 (5.9)	0	–	NINCDS-ADRDA, MMSE	Present teeth, DMFT
Syrjala et al. [51], Finland	Cross-sectional	49 AD 16 VaD 11 OD	84.8 (5.6) 82.2 (4.7) 85.3 (4.8)	278	81.4 (4.6)	DSM-IV, McKeith, DSM-III-R	Present teeth, dentures
Warren et al. [52], USA	Case-control	45 AD 52 OD	81.6 (6.9) 81.4 (7.3)	133	80.3 (6.8)	MMSE, chart, NT, scans	Dental habits, present teeth, dentures, coronal caries, root caries
Zenthöfer et al. [53], Germany	Case-control	57	83.1 (10.6)	36	82.6 (9.0)	MMSE, medical chart	Decayed and missing teeth
Zenthöfer et al. [54], Germany	Cohort	33	81.7 (9.0)	60	83.4 (10.4)	MMSE, medical chart	Missing teeth

*AD* Alzheimer dementia, *ADJCI* adjusted caries increments, *ADL* Activities of Daily Living, *CASI* Cognitive Abilities Screening Instrument, *CCI* crude caries increment, *CDR* Clinical Dementia Rating, *CERAD* Consortium to Establish a Registry for Alzheimer’s Disease, *COD* classification of dementia, *CT* computer tomography, *DMFT* decayed missing filled teeth, *DQ* Dementia Questionnaire, *DSM* Diagnostic and Statistical Manual of Mental Disorders, *GOHAI* Geriatric Oral Health Assessment Index, *ICD* International Classification of Diseases, *McKeith* consensus criteria for Dementia with Lewy Bodies by McKeith, *MDS-COGS* Minimum Data Set Cognition Scale, *MHLW* Ministry of Health, Labour, and Welfare, *MiD* mild dementia, *MoD* moderate dementia, *MMSE* Mini Mental State Examination, *MRI* Magnetic Resonance Imaging, *mv* missing value, *NCI* net caries increment, *ND* no dementia, *NINCD-ADRDA* National Institute of Neurological Disorders and Stroke Alzheimer’s Disease and Related Disorders Association, *NOS* Newcastle-Ottawa Scale, *NP* Neuropsychological Examination, *NT* Neurological Testing, *OD* other dementia, *PCR* Plaque Control Record, *PET* Positron Emission Tomography, *SeD* severe dementia, *VaD* vascular dementia

Altogether, the included studies about dental hard tissues involved 3770 participants with dementia and 4036 participants without dementia. The mean age of the participants with dementia was 78.18, and the mean age of the participants without dementia was 74.0 years. The reported method to classify the group of people with dementia varied. Seven studies specified the dementia subtype: Alzheimer’s disease, vascular dementia, and other types of dementia, such as Lewy bodies [30, 35, 36, 38, 42, 51, 52]. Three studies divided the group according to dementia severity [26, 28, 41]. Four studies were about nursing home residents (Table 2), without separate data about older people with and without dementia [29, 56–58]. The authors of these studies (Chalmers et al. and Hopcraft et al.) were contacted, but it was impossible to obtain separate data for the participants with and without dementia.

**Table 2:** Overview of studies about the oral health in nursing home residents, including people with dementia, without subdivision in people with dementia and without dementia

Study	Design	NHR (=N)	Mean age	Percentage dementia	Dementia	Oral health
Chalmers et al. [14, 29]	Cross-sectional (baseline)	224	83.2	75.0 %	MMSE	Dental habits, present teeth, dentures, DMFT, coronal caries, root caries, retained roots
Chalmers et al. [56]	Longitudinal cohort (follow-up)	224	83.2	>65.0 %	MMSE	Orofacial pain, dental habits, present teeth, DMFT, coronal caries, root caries, retained roots
Chalmers et al. [57]	Longitudinal cohort (comparison)	224	83.6	63.4 %	MMSE	Orofacial pain, dental habits, CCI, NCI, ADJCI
Hopcraft et al. [58]	Cross-sectional	510	mv	38.0 %	Chart	Present teeth, DMFT, coronal caries, retained roots

*ADJCI* Adjusted caries and filling increments, *ADSNH* Adelaide Dental Study of Nursing Homes, *CCI* crude caries increment, *DMFT* decayed missing filled teeth, *MMSE* Mini Mental State Examination, *mv* missing value, *NCI* net caries increment, *NHR* nursing home residents

### Group and outcome variables

Dementia was classified (Table 1) with the Diagnostic and Statistical Manual of Mental Disorders (DSM-III or IV) [60, 61] or International Classification of Disease (ICD-10) [62]; National Institute of Neurological and Communicative Disorders and Stroke and the Alzheimer’s disease and Related Disorders Association (NINCDS-ADRDA) [63, 64]; computed tomography (CT); Magnetic Resonance Imaging (MRI); Positron Emission Tomography (PET) [65]; Clinical Dementia Rating (CDR) [66]; classification of dementia by the Ministry of Health, Labour, and Welfare (MHLW) of Japan [46]; and/ or the existing medical chart of the participant. In addition to dementia diagnosis, measurements for cognitive status were used, such as the Abbreviated Mental Test (AMT) [28, 67], Mini-Mental State Examination (MMSE) [68], or Minimum Data Set Cognitive Score (MDS-COGS) [33, 69]. Additionally, functional measures (e.g., Activities of Daily Living) were used.

The studies showed a variety of outcome measures concerning dental hard tissues (Table 1). The most used measures were number of teeth present [17], DMFT index [70–72], number of retained roots, and number of teeth with coronal and root caries. The development of dental caries was measured using the following outcome measures: crude caries increment (CCI) [18, 36], root caries index (RCI) [3], net caries increment (NCI) [18, 36], and adjusted caries and filling increments (ADJCI) [18, 36]. The use of prosthetics was reported by percentages of edentulousness and presence of removable prosthetics.

### Quality assessment

An overview of the results of the quality assessment with the Newcastle-Ottawa Scale [13] is presented in Tables 3, 4, 5, and 6. The NOS scores of the assessed articles ranged from 1 to 9; the median score was 5 and the mean was 4.9 (SD 2.2). Of the 37 studies, 9 studies had an NOS score of 7 or higher.

**Table 3:** Methodological quality assessment of the included cohort studies with the Newcastle-Ottawa Scale

	Selection				Comparability		Outcome			Score
Cohort study	Representativeness of cases	Selection of controls	Ascertainment of exposure	Demonstration outcome of interest not present at start of study	Age	Gender	Assessment of oral health	Follow up long enough	Adequacy of follow up	Total
Chalmers et al. [14]	*+*	*−*	*−*	*−*	+	+	+	+	−	5
Chalmers et al. [15]	+	−	−	+	+	+	+	+	+	7
Chalmers et al. [56] NHR	+	−	−	−	−	−	+	+	−	3
Chalmers et al. [57] NHR	+	−	−	?	−	−	+	+	−	3
De Souza Rolim et al. [34]	+	−	+	−	−	−	+	−	−	3
Ellefsen et al. [36, 37]	+	+	+	−	+	+	+	+	−	7
Hatipoglu et al. [42]	−	−	−	+	−	−	+	+	?	3
Jones et al. [43]	+	−	−	+	+	+	+	+	−	6
Ship and Puckett [12]	+	+	+	−	+	−	+	+	−	6
Sumi et al. [50]	+	−	+	−	−	−	+	+	?	4
Zenthöfer et al. [54]	+	+	+	+	+	+	+	+	+	9

*+* met, *−* unmet, *?* unclear

**Table 4:** Methodological quality assessment of the included case-control studies with the Newcastle-Ottawa Scale

	Selection				Comparability		Exposure			Score
Case-control study	Definition of cases	Representativeness of cases	Selection of controls	Definition of controls	Age	Gender	Assessment of oral health	Same method cases and controls	Non-response rate	Total
Chu et al. [32]	*−*	*+*	*−*	*+*	+	+	+	−	−	5
De Souza Rolim et al. [34]	+	+	+	+	+	+	+	+	−	8
Kossioni et al. [44]	+	+	−	−	+	+	+	+	−	6
Nordenram et al. [47]	+	+	+	+	+	+	+	+	−	8
Warren et al. [52]	+	+	−	−	+	+	+	+	+	7
Zenthöfer et al. [53]	−	−	+	−	+	−	+	+	−	4

*+* met, *−* unmet, *?* unclear

**Table 5:** Methodological quality assessment of the included cross-sectional studies with the Newcastle-Ottawa Scale

Cross-sectional study	Selection				Comparability		Exposure			Score
	Definition of cases	Representativeness of cases	Selection of controls	Definition of controls	Age	Gender	Assessment of oral health	Same method cases and controls	Non-response rate	Total
Adam and Preston [28]	−	+	+	−	−	−	+	−	?	3
Bomfim et al. [24]	−	−	+	−	−	−	?	−	?	1
Chalmers et al. NHR [14, 29]	−	+	+	−	−	−	+	+	?	4
Chapman and Shaw [30]	−	+	−	−	−	−	+	−	−	2
Chen et al. [22, 31]	+	+	+	+	−	−	+	+	−	6
Cohen-Mansfield [33]	−	−	−	−	−	−	+	−	−	1
Ellefsen et al. [35]	+	+	+	−	+	+	+	+	−	7
Ellefsen et al. [38]	+	+	+	−	+	+	+	+	−	7
Elsig et al. [39]	+	+	+	−	−	−	+	+	−	5
Eshkoor et al. [40]	−	+	−	−	−	−	−	−	−	1
Furuta et al. [41]	−	+	−	−	+	+	+	+	−	5
Hopcraft et al. NHR [58]	−	+	+	−	−	−	+	+	−	4
Lee et al. [26]	+	−	−	+	+	−	+	+	?	5
Luo et al. [45]	+	+	+	+	−	+	−	+	+	7
Minakuchi et al. [46]	+	+	−	+	+	−	+	+	−	6
Philip et al. [5, 16]	−	−	−	+	−	−	+	+	−	3
Ribeiro et al. [48]	+	+	−	+	−	−	+	+	−	5
Srisilapanan et al. [49]	−	+	−	−	−	−	+	−	−	2
Syrjala et al. [51]	+	+	+	+	+	+	+	+	−	8

*+* met, *−* unmet, *?* unclear

**Table 6:** Methodological quality assessment of the included randomized clinical trial with the Newcastle-Ottawa Scale

RCT	Selection				Comparability		Exposure			Score
	Definition of cases	Representativeness of cases	Selection of controls	Definition of controls	Age	Gender	Assessment of oral health	Same method cases and controls	Non-response rate	Total
Fjeld et al. [25]	+	−	+	+	?	?	+	+	+	7

*+* met, *−* unmet, *?* unclear

In 14 (=53.8 %) of the non-cohort the studies, the DSM, ICD, or NINCDS-ADRDA was used for the classification of the dementia diagnosis. For 30 (=81.1 %) studies, the participants demonstrated good representativeness of the classification “older people with dementia.” Controls, in this case older people without dementia, often (=54.1 %) came from other sources than the cases. In only 11 (=29.7 %) of the non-cohort studies, it was explicitly stated that the controls had no history of dementia. Of all 37 studies, 51.4 % had comparable age and 37.8 % had comparable gender between cases and controls. Almost all studies (=91.9 %) used a standardized, structured method for the dental examination. Only 3 studies (=18.2 % of the non-cohort studies) described the non-response rate [25, 45, 52]. For most of the 11 cohort studies (=90.9 %), the follow-up period was longer than 3 months. At the same time, the number of subjects lost to follow-up was reported in only two (=22.2 %) of the cohort studies.

### Results for each outcome variable

With respect to edentulousness, a wide range of percentages between studies was seen among older people with and without dementia (Table 7). For people without dementia, percentages varied from 14.0 to 70.0 % [28, 32] and for older people with dementia from 11.6 to 72.7 % [51, 49].

**Table 7:** Edentulousness in older people with and without dementia

Study	Number of participants Mean age in years (SD)		Edentulousness		Specification
	No dementia	Dementia	No dementia	Dementia	
Adam and Preston [28]	54 ND-MiD 85.5 (7.6)	81 MoD-SeD 80.8 (7.6)	ND-MiD 70.0 %	MoD-SeD 63.0 %	Not dentate
Bomfim et al. [24]	mv	mv	46.7 %	40.0 %	No specification
Chapman and Shaw [30]	0	85 AD 74.9	–	AD 64.7 %	No teeth, with and without dentures
Chu et al. [32]	59 79.8 (7.4)	59 79.8 (7.4)	14.0 %	17.0 %	Not dentate
De Souza Rolim et al. [34]	30 61.2 (11.2)	29 75.2 (6.7)	43.3 %	32.3 %	*p* = .614
Elsig et al. [39]	22 81.9 (6.5)	29 82.5 (6.3)	54.6 %	62.1 %	*p* = .774
Kossioni et al. [44]	84	27 76.5 (6.8)	–	62.9 %	No teeth, with and without dentures
Nordenram et al. [47]	40 87 (6.6)	40 AD 87 (7.0)	43.0 %	MoD 36.0 % SeD 45.0 %	No teeth, with and without dentures
Srisilapanan et al. [49]	0	69 75.5 (7.0)	–	11.6 %	No teeth
Syrjala et al. [51]	278 81.4 (4.6)	49 AD 84.8 (5.6)	44.6 %	AD 63.3 %	No teeth, with and without dentures
		16 VaD 82.2 (4.7)		VaD 68.8 %	
		11 OD 85.3 (4.8)		OD 72.7 %	
Warren et al. [52]	133 ND 80.3 (6.8)	45 AD 81.6 (6.9) 52 OD 81.4 (7.3)	31.6 %	AD 40.0 % OD 32.0 %	No specification

*AD* Alzheimer dementia, *Dem* dementia, *DQ* Dementia Questionnaire, *MiD* mild dementia, *MoD* moderate dementia, *mv* missing value, *ND* no dementia, *OD* other dementia, *SeD* severe dementia, *VaD* vascular dementia

In terms of denture utilization, there was also a great variation among older people with and without dementia (Table 8). For older people without dementia, percentages ranged from 17.0 to 81.8 % [47, 73]; for older people with dementia, this number ranged from 5.0 to 100.0 % [42, 47]. The lowest percentage (5.0 %) was seen in a group of people with severe dementia (MMSE score below 10) [47].

**Table 8:** Dentures in older people with and without dementia

Study	Number of participants Mean age in years (SD)		Dentures	
	No dementia	Dementia	No dementia	Dementia
Bomfim et al. [24]	mv	mv	20.0 %	20.0 %
Chalmers et al. [15]	113 <79: 88 80+: 25	103 <79: 82 80+: 21	27.6–30.1 %	20.7–23.3 %
Chapman and Shaw [30]	0	85 AD 74.9 (mv)	–	59.0 %
Chen et al. [22, 31]	–	51 community living 79.3 (8.0)	–	Community living 48.0 %
		18 assisted living 80.9 (12.6)		Assisted living 38.9 %
		501 nursing home residents 82.6 (9.6)		Nursing home residents 47.1 %
De Souza Rolim et al. [34]	30 61.17 (11.2)	29 75.17 (6.7)	43.3 %	25.8 %
Eshkoor et al. [40]	71 (mv)	1210	81.8 %	86.2 %
Hatipoglu et al. [42]	47 65.3 (7.0)	31 AD 67.6 (9.1)	*Maxillary* 57.0 % *Mandibular* 55.0 %	*Maxillary* AD 97.0 % *Mandibular* AD 100.0 %
Kim et al. [59]	919	0	53.0 %	–
Nordenram et al. [47]	40 87 (6.6)	40 AD 87.0 (7.0)	17.0 %	MoD 7.0 % SeD 5.0 %
Ship and Puckett [12]	21 65 (12)	21 AD 64.0 (9.0)	43.0 %	AD 40.0–67.0 %
Srisilapanan et al. [49]	0	69 75.5 (7.0)	–	40.6 %
Syrjala et al. [51]	278 ND 81.4 (4.6)	49 AD 84.8 (5.6)	73.7 %	AD 75.5 %
		16 VaD 82.2 (4.7)		VaD 68.6 %
		11 OD 85.3 (4.8)		OD 72.2 %

*AD* Alzheimer’s disease, *Dem* dementia, *MoD* moderate dementia, *mv* missing value, *ND* no dementia, *NHR* nursing home residents, *OD* other dementia, *SeD* severe dementia, *VD* vascular dementia

The number of teeth present was the most commonly used indicator for dental health, and there was a wide range within both groups (Table 9). For people without dementia, it varied between 2.0 and 20.2 [24, 37], and for people with dementia, it varied between 1.7 and 20.0 [51, 49].

**Table 9:** Number of present teeth in older people with and without dementia

Study	Number of participants Mean age in years (SD)		Number of present teeth		No dementia vs dementia *p* value
	No dementia	Dementia	No dementia	Dementia	
Bomfim et al. [24]	mv	mv	2.0 (8.5)	3.0 (3.7)	mv
Chalmers et al. [14, 15]	116 <79: 78.4 % 80+: 21.6 %	116 <79: 78.4 % 80+: 21.6 %	17.2 (mv)	18.0	>.05
Chapman and Shaw [30]	–	85 AD 74.9	–	12.8	n/a
Chen et al. [31]	–	51 community 79.3 (8.0)	–	Community living 18.2 (7.2)	n/a
		18 assisted 80.9 (12.6)		Assisted living 19.3 (6.8)	
		501 NHR 82.6 (9.6)		Nursing home residents 17.4 (7.9)	
Ellefsen et al. [36]	13 79.9 (7.7)	49 AD 83.6 (5.5)	20.2 (8.9)	AD 17.3 (7.4)^a^	≤.001 for AD
		15 OD 81.3 (4.0)		OD 16.1 (9.0)	
Ellefsen et al. [38]	–	61 AD 82.8 (5.7)	–	AD 16.5 (7.4)	n/a
Elsig et al. [39]	22 81.9 (6.5)	29 82.5 (6.3)	6.5 (8.8)	4.9 (8.3)	.533
Fjeld et al. [25]	43 88.5 (6.6)	159 85.5 (7.7)	20.1 (6.1)	20.0 (5.8)	mv
Hopcraft et al. [58]		510 NHR 194 Dem	14.6 (0.7)	(0.7)	>.05
Jones et al. [43]	46 66.1 (6.9)	23 67.4 (7.5)	18.2 (7.5)	AD 17.9 (8.1)	.90
Kossioni et al. [44]	84	27 76.5 (6.8)	–	4.4 (7.2)	n/a
Ribeiro et al. [48]	30 67.8 (5.4)	30 79.1 (5.6)	Median 13.5^a^ (0.0–28.0)	Median 1.0^a^ (0.0–22.0)	.0004
Srisilapanan et al. [49]	–	69 75.5 (7.0)	–	19.5 (8.4)	n/a
Sumi et al. [50]	–	10 77.7 (5.9)	–	12.7	n/a
Syrjala et al. [51]	278 81.4 (4.6)	49 AD 84.8 (5.6)	15.0 (8.2)	AD 10.9 (7.0)	
		16 VaD 82.2 (4.7)		VaD 7.8 (3.8)	
		11 OD 85.3 (4.8)		OD 1.7 (1.2)	
Warren et al. [52]	133 80.3 (6.8)	45 AD 81.6 (6.9)	13.0 (10.8)	AD 10.0 (10.1)	*p* > .05
		52 OD 81.4 (7.3)		OD 13.0 (10.6)	

*AD* Alzheimer dementia, *ADS NH* Adelaide Dental Study of Nursing Homes, *Dem* dementia, *MiD* mild dementia, *MoD* moderate dementia, *mv* missing value, *N/A* not applicable, *ND* no dementia, *NHR* nursing home residents, *OD* other dementia, *OH CLOAD* oral health of community-living older adults with dementia, *SeD* severe dementia, *VaD* vascular dementia

^a^Significant difference between groups

The DMFT index (Table 10) was 19.7 to 26.1 in people without dementia [5, 42], and 14.9 to 28.0 [48, 49] in people with dementia. The lowest DMFT was 14.9, which was derived from a cross-sectional study from Thailand examining older people with dementia without using a control group [49]. Only five studies compared older people with and without dementia, and just one study found a significant difference between the two groups; DMFT 25.5 in people without and DMFT 28.0 in people with dementia [48].

**Table 10:** Decayed, missing, and filled teeth and DMFT index in older people with and without dementia

Study	Number of participants Mean age in years (SD)		Decayed		Missing		Filled		DMFT	
	No dementia	Dementia	No dementia	Dementia	No dementia	Dementia	No dementia	Dementia	No dementia	Dementia
Adam and Preston [28]	54 ND-MiD 85.5 (7.6)	81 MoD-SeD 80.8 (7.6)	1.1 (3.4)	0.80 (1.9)	28.2 (6.6)	27.3 (7.7)	ND-MiD 0.7 (1.3)	0.90 (2.4)		
Chalmers et al. [14]	116 <79 years: 91 80+: 25	116 <79: 91 80+: 25	0.0–0.4	0.5–1.6*	–	–	24.7–25.7	22.1–23.9	–	–
Chalmers et al. [15]	113 <79 years: 88 80+: 25	103 <79: 82 80+: 21	0.0–0.1	0.3–1.3*	–	–	–	–	–	–
Chapman and Shaw [30]	0	85 AD 74.9	–	1.4 (0.3)	–	17.8 (1.0)	–	6.4 (0.7)	–	25.6 (0.7)
Chen et al. [22, 31]	–	51 community 79.3 (8.0)	–	Community 5.5 (5.4)	–	–	–	Community 10.4 (6.3)	–	–
		18 assisted 80.9 (12.6)		Assisted 5.3 (4.1)				Assisted 10.9 (6.0)		
		501 NHR 82.6 (9.6)		NHR 6.0 (5.2)				NHR 8.7 (6.3)		
Chu et al. [32]	59 79.8 (7.4)	59 79.8 (7.4)	0.8 (1.4)	1.2 (1.9)	18.3 (8.9)	18.9 (9.4)	2.4 (2.5)	2.5 (3.3)	21.5 (8.2)	22.3 (8.2)
De Souza Rolim et al. [34]	30 61.2 (11.2)	29 75.2 (6.7)	–	–	–	–	–	–		27.2 (5.7) Range 11–32
Hatipoglu et al. [42]	47 65.3 (7.0)	31 AD 67.6 (9.1)	–	–	–	–	–	–	19.7 (9.5)	24.2 (6.8)
Hopcraft et al. [58]	316 of 510 NHR	194 of 510 NHR	2.9 (0.4)	2.4 (0.3)	17.4 (0.7)	17.9 (0.7)	4.8 (0.6)	4.8 (0.6)	25.0 (0.4)	25.0 (0.5)
Kossioni et al. [44]	Other psychotic diagnosis	27 76.5 (6.8)	–	1.8 (2.9)	–	–	–	0.9 (1.5)	–	–
Lee et al. [26]	169 77.4 (5.8)	19 MiD 83.9 (7.9)	CC + RC	CC + RC	12.7 (7.6)	10.2 (7.5)	–	–	–	–
Luo et al. [45]	2389 70.0 (7.7)	120 80.9 (7.4)	–	–	9.3	18.7**	–	–	–	–
Philip et al. [5]	102 84.3 (9.9)	84 85.7(9.6)	2.9 (3.0)	3.0 (3.9)	18.0 (7.1)	17.4 (7.3)	5.0 (4.8)	5.3 (5.0)	26.1 (4.2)	25.9 (4.5)
Ribeiro et al. [48]	30 67.8 (5.5)	30 79.1 (5.6)	–	–	–	–	–	–	25.5 (12.0–28.0)*	28.0 (22.0–28.0)*
Srisilapanan et al. [49]	0	69 75.5 (7.0)	–	1.5 (2.3)	–	12.6 (8.4)	–	0.8 (1.9)	–	14.9 (9.2)
Zenthöfer et al. [53]	36 82.6 (9.0)	57 83.1 (10.6)	0.7 (1.4)	0.6 (1.3)	19.9 (9.1)	20.8 (8.5)	–	–	–	–
Zenthöfer et al. [54]	60 83.4 (10.4)	33 81.7 (9.0)	–	–	20.5 (8.5)	20.5 (9.2)	–	–	–	–

*AD* Alzheimer’s disease, *CC* coronal caries, *D* decayed, *Dem* dementia, *DMFT* decayed missing filled teeth, *F* filled, *M* missing, *MoD* moderate dementia, *ND* no dementia, *NHR* nursing home residents, *OD* other dementia, *RC* root caries, *SeD* severe dementia, *VaD* vascular dementia

*p < .05, **p < .001

Taking the DMFT categories separately, “decay” varied from 0.0 to 2.9 in the group of older people without dementia [14, 15, 58] and 0.3 to 6.0 in the group of older people with dementia [15, 31], “missing” from 9.3 to 28.2 in the group without dementia [28, 45] and 10.2 to 27.3 in the group with dementia [26, 28], and “filled” from 0.7 to 25.7 in the group without dementia [14, 28] and 0.8 to 23.9 in the group with dementia [14, 49].

The reviewed studies showed that older people with dementia had more coronal caries (0.1–2.9) [35, 38, 52] than older people without dementia (0.0–1.0) [14, 15, 35, 38]. In addition, older people with dementia had more root caries (0.6–4.9) [35, 38, 52] than people without dementia (0.3–1.7) [14, 15, 35, 38]. Furthermore, retained roots were more common in people with dementia (0.2–10) [14, 35] than in people without dementia (0.0–1.2) [5, 35]. (Table 11).

**Table 11:** Retained roots, root caries, and coronal caries in older people with and without dementia

Study	Number of participants Mean age in years (SD)		Coronal caries Mean number (SD)		Root caries Mean number (SD)		Retained roots Mean number (SD)	
	No dementia	Dementia	No dementia	Dementia	No dementia	Dementia	No dementia	Dementia
Chalmers et al. [14] Chalmers et al. [15]	116 <79: 91 80+: 25	116 <79: 91 80+: 25	0.0*	0.5*	0.3*	0.8*	*Decayed* 0.0* *Sound* 0.1	*Decayed* 0.3* *Sound* 0.2
De Souza Rolim et al. [34]	30 61.2 (11.2)	29 75.2 (6.7)	3.4 %	6.8 %	–	–	10.2 %	6.8 %
Ellefsen et al. [35] Ellefsen et al. [38]	19 79.8 (7.3)	61 AD 82.8 (5.7)	1.0*	2.9*	1.7*	AD 4.9*	0.0*	AD 10.0*
		26 OD 81.5 (4.8)				OD 2.3*		OD 0.5*
Jones et al. [43]	46 66.1 (6.9)	23 AD 67.4 (7.5)	0.8	1.4	0.4	1.8	–	–
Lee et al. [26]	169 77.4 (5.8)*	19 MiD 83.9 (7.9)*	0.8 (2.1)	1.0 (1.6)	0.5 (1.1)*	1.8 (3.6)*	–	–
Philip et al. [5]	102 84.3 (9.9)	84 85.7 (9.6)	–	–	–	–	1.2 D 0.9	1.8 D 1.4
Warren et al. [52]	133 ND 80.3 (6.8)	45 AD 81.6 (6.9) 52 OD 81.4 (7.3)	0.4	AD 0.1 OD 0.4	0.8	AD 0.6 OD 0.6	–	–

*AD* Alzheimer’s disease, *D* decayed, *Dem* dementia, *MiD* mild dementia, *ND* no dementia, *OD* other dementia, *VaD* vascular dementia

*p < .05

Although dental hard tissues can be an important source of orofacial pain, only seven of the included studies published data about the presence of orofacial pain [15, 19, 28, 33, 34, 44, 74]. The presence of reported dental pain in older people with dementia varied between 7.4 and 21.7 %. Only in the study of Cohen-Mansfield and Lipson, pain with dental etiology was the central research question [33]. In this study, 60.0 % of the assessed participants were considered to have a dental pain-causing condition (Table 12). For older people without dementia, the orofacial pain prevalence was 6.7–18.5 % [28, 34].

**Table 12:** Orofacial pain in older people with and without dementia

Study	Number of participants Mean age in years (SD)		Orofacial pain		Pain measurement
	No dementia	Dementia	No dementia	Dementia	
Adam and Preston [28]	54 ND-MiD 85.5 (7.6)	81 MoD-SeD 80.8 (7.6)	18.5 %	7.4 %	Questionnaire: presence or absence of pain in the last 4 weeks, asked to individuals and/ or caregivers; nearly 60 % of the responses attained from caregivers
Chalmers et al. [15]	113 <79: 88 80+: 25	103 <79: 82 80+: 21	11.2–11.5 %	18.4–19.0 %	Questionnaire: current pain or discomfort. Asked to guardian/caregiver if necessary
Cohen-Mansfield [33]	–	21 88.0 (mv)	–	60.0 %	Dental exam: considered to have pain-causing conditions according to dentist
De Souza Rolim et al. [34] De Souza Rolim et al. Evaluation^a^ [34]	30 61.17 (11.2)	29 75.17 (6.7)	6.7 %	20.7 %	Questionnaire and dental exam: orofacial pain characteristics and Visual Analog Scale, McGill Pain Questionnaire
Kossioni et al. [44]	–	23 76.3 (7.1)	–	21.7 %	Questionnaire: pain when chewing

*Dem* dementia, *MiD* mild dementia, *MoD* moderate dementia, *mv* missing value, *SeD* severe dementia

^a^Same data as de Souza Rolim [34]

The heterogeneity, specifically the clinical and methodological variability, between the studies was considered too large to perform a meta-analysis.

## Discussion

This is the first systematic review with a quantitative overview of oral health variables in older people with dementia, compared to older people without dementia. Several qualitative reviews already stated the importance of good oral health in older people with dementia [75–83]. This review summarizes that the number of teeth present is comparable between older people with dementia and cognitively intact older people [14, 15, 25, 39, 43, 51, 52, 58]. The number of teeth present was the most commonly used measure for dental health, presumably because of its simplicity.

Studies that compare older people with and without dementia, showed similar, high DMFT scores for both groups [5, 32, 42, 48, 58]. Although the DMFT index gives an indication of the dental caries history as a whole, it does not distinguish between decayed, missing, and filled teeth separately. To get a better indication of disease and treatment need, the presence of caries should be assessed individually. Dental decay can be divided in coronal and root caries, which is a valuable distinction, considering the etiology and treatment methods of these types of caries. Coronal caries and root caries are significantly more common in older people with dementia than in those without dementia. This difference can be explained by cognitive, medical, and functional changes in people with dementia. For example, agitated behavior, characteristic for dementia, may complicate oral care [84], resulting in increased plaque accumulation and higher risk of caries [14]. In addition, reduced cooperation with dental treatment may constrain the possibilities of dental treatment [85]. The risk of caries increases even further, as a result of decreased submandibular saliva flow rates in people with Alzheimer’s disease [86], and changes in food composition (e.g., more sticky, grinded, and cariogenic food), which are often seen in people with dementia [39, 58]. Furthermore, functional changes in dementia, like declined handgrip and motor skills, play a role in the caries risk [39, 48]. More specifically, the decline in motor coordination might result in more difficulty performing oral care [48] and lower chewing and swallowing efficiency [39]. Remarkably, studies looking at coronal and root caries separately show significantly more caries in older people with dementia. One explanation is that some studies did not include root caries as decay in the DMFT index, as this was not mentioned in all articles [28, 42, 48, 87].

Retained roots are more present in older people with dementia than older people without dementia. This may be a result of the higher caries prevalence, fewer dental checks, resistance-to-care behavior, and decreased verbal communication skills [88, 89]. Lee and colleagues stated that, in the USA, people with dementia are less likely to visit the dentist regularly and the last visit to the dentist was a longer time ago, compared to older people without cognitive impairment [88]. Furthermore, an article about the barriers to good oral hygiene in nursing homes pointed out that resistance-to-care behavior is a major threshold in providing good oral care, which can be overcome by education of health workers and more time to provide oral care [90]. Additionally, verbal communication about dental problems and pain can be complicated in people with dementia, because of the short-term memory loss and language disturbances, like aphasia [91].

For edentulousness, the wide range in percentages might have been related to cultural differences [92, 93] and the small number of studies and participants. For instance, people in different countries have different diets, oral hygiene habits, and access to professional dental care [3, 94].

Dentures were worn by approximately the same percentage of older people either with or without dementia [12, 15, 51]. However, one study examined people in different stages of dementia and found lower percentages of denture use in people with more severe dementia [15, 47]. Adam and Preston suggest that “the high rate of not wearing dentures in the moderate/severe dementia group may in part be due to the dementia itself” [28]. A decrease of denture use with the progress of dementia could be explained by the lower tolerance of dentures, decreased control of oral musculature, decreased quality and quantity of saliva, and/ or higher risk of denture loss [85, 95]. Additionally, as people are edentulous for a longer time, the processus alveolaris resorbs more, resulting in a decrease of denture retention, especially in the lower jaw [96]. This increases the risk of aspiration of the lower dentures, particularly in older people with dementia, who are at increased risk of aspiration of foreign material [97].

Strikingly, orofacial pain in older people with dementia (7.4–21.7 %) was rarely studied [15, 33]. This is interesting, because this particular group seems to be at higher risk for this type of pain, considering the higher prevalence of oral health problems and the loss of verbal communication skills as the dementia progresses. Even more so, because being free of pain is considered an important factor in quality of life [1].

### Strengths and limitations

The main strengths of this review are its systematic approach, the quality assessment of the articles, the quantitative overview of the dementia and oral health variables, and the involvement of a multidisciplinary team, including a neuropsychologist, dentists, and a pain specialist. For the search, there were no language limitations. Next to the described search, additional searches were done with the search terms facial pain, dental pain, DMFT, caries, and teeth present, in combination with dementia, to check the completeness of the results of the original search. Regarding the quality of the studies, most have a good, representative selection of cases and controls, a good comparability between the groups, and a systematic approach of the dental examination.

Limitations of this review are that the included studies showed a variety in outcome measures, not all included studies reported the standard deviations of the published mean values, and some studies about nursing home residents did not distinguish between older people with and without dementia. In addition, the number of RCTs was small, the number of high quality studies was low, and the heterogeneity was too large to perform a meta-analysis. Within the studies, the non-response and follow-up rate of the participants was often insufficiently described. In order to enable a better interpretation, it is important that these results are published. Despite the mentioned limitations, in this review the outcome measures, standard deviations and means, classification of dementia, and NOS scores of the studies are represented in a systematic manner to enhance a better interpretation of the different studies.

When looking at the effect of the quality on the studies, the main thing that strikes is the higher amount of coronal and root caries in older people without dementia in high quality studies [52], compared to all studies. Furthermore, the amount of retained roots in older people with dementia is the highest in the only high-quality study that compares retained roots in older people with and without dementia [35]. When only the high-quality studies are considered, the percentage of orofacial pain in older people with dementia is higher [15]. The ranges of outcome values get smaller when solely looking at the higher quality studies, especially for edentulousness [51, 52], denture use [47, 51], and the number of teeth present [37, 51, 52]. This seems logical, considering the smaller amount of studies involved.

### Considerations and suggestions

This study shows a broad range of methods to classify the group of people with dementia. The MMSE is most commonly used, even though it is only a short cognitive screening instrument and not suitable for dementia diagnosis [98]. The advantages of the MMSE are its easy and quick application and the possibility of using this tool in moderate stages of dementia (from MMSE 14), where more extensive neuropsychological testing is no longer possible [68]. To diagnose dementia, extensive diagnostic examination should take place, and structural classification with systems like the ICD and DSM are preferred [61, 99, 100]. To distinguish between dementia subtypes, neuroimaging is a valuable addition [101].

For oral health, a broad range of methods is also seen, with the number of teeth present being the most common variable studied. While the number of teeth present is easy to measure and compare between studies, it does not specify the state of the teeth. The DMFT also provides information about the presence of caries and fillings in the teeth and is a widely used method, which enables comparing results between studies [102]. However, the method was developed in 1930 for epidemiological research in children [103] and seems unsuitable for present-day dentistry in people, which includes implants, crowns, and bridges. Further limitations of the DMFT are that teeth can be lost for reasons other than caries; it cannot be used to assess root caries; and it gives equal weight to decayed, missing, and filled teeth [104]. There is a need for an international, standardized method for dental examination in (older) people, dealing with the limitations stated above. Suggested items for the examination of dental hard tissues are the number of teeth present and the presence of implants, crowns, bridges, fillings, coronal caries, root caries, and retained roots. To investigate the chewing efficiency, Elsig and colleagues also suggested to include a chewing efficiency test into a standard examination [39]. In addition, the soft tissues should be examined. Suggestions for the examination of the dental soft tissues are beyond the scope of this article and will be discussed in a separate review.

With regard to oral health in older people with dementia, Chalmers and colleagues already suggested to examine the possible relationship between dental problems, dental pain, and challenging behavior in older people with dementia [14]. As of yet, this relationship is still scarcely studied, although dental discomfort might be an underlying cause of behavioral problems [105, 106]. This issue may even be more urgent for people with vascular dementia, in whom the pain experience is suggested to be increased, due to the presence of white matter lesions [107, 108]. However, the prevalence of orofacial pain in dementia subtypes has not been studied yet and is a suggested subject for future research.

## Conclusion

In conclusion, this systematic review found that older people with dementia have worse overall oral health than older people without dementia, including coronal caries, root caries, and retained roots. In contrast, they had an equivalent number of teeth present, similar rate of edentulousness, and equivalent decayed missing filled teeth index. Unfortunately, few studies have focused on orofacial pain in older people with dementia. Oral health, and specifically orofacial pain in older people with dementia, is in dire need of further attention.
